# Electroacupuncture Improves Intestinal Motility through Exosomal miR-34c-5p Targeting SCF/c-Kit Signaling Pathway in Slow Transit Constipation Model Rats

**DOI:** 10.1155/2022/8043841

**Published:** 2022-09-12

**Authors:** Hongjun Kuang, Chengshun Zhang, Wei Zhang, Huzhi Cai, Layuan Yang, Nan Yuan, Yangyang Yuan, Yutao Yang, Chuanyi Zuo, Feng Zhong

**Affiliations:** ^1^The First Hospital of Hunan University of Chinese Medicine, Changsha 410007, Hunan Province, China; ^2^Acupuncture and Tuina School-Third Teaching Hospital, Chengdu University of Traditional Chinese Medicine, Chengdu 610075, Sichuan Province, China; ^3^Department of Acupuncture, Chongqing Traditional Chinese Medicine Hospital, Chongqing 400021, China

## Abstract

**Background:**

The pathogenesis of slow transit constipation (STC) is associated with exosomal miR-34c-5p. Electroacupuncture (EA) improves gastrointestinal motility in gastrointestinal disorders, especially STC. Our study aimed to explore the mechanism by which EA improves intestinal motility by modulating the release of exosomes and the transmission of exosomal miR-34c-5p.

**Methods:**

Fifty rats were randomly divided into five groups. STC model rats were induced, and GW4869, the exosome release inhibitor, was used to inhibit the release of exosome. The serum exosomes were authenticated under a transmission electron microscope and nanoparticle tracking analysis. RT-qPCR detected the expression of miR-34c-5p in serum exosomes and colonic tissues. The fecal number in 24 hours, Bristol scores, and intestinal transit rates were used to assess intestinal motility. Subsequently, hematoxylin and eosin (H&E) staining was used to examine the colonic mucosal histology. Finally, the expression of stem cell factor (SCF) and receptor tyrosine kinase (c-Kit) protein was measured using immunohistochemistry staining.

**Results:**

We found that EA upregulated exosomal miR-34c-5p in serum and downregulated miR-34c-5p in colonic tissues (*P* < 0.01). EA improved fecal numbers in 24 hours, Bristol scores, and intestinal transit rates in STC rats (*P* < 0.01). EA recovered the colonic histological structure and enhanced the expression of SCF and c-Kit protein (*P* < 0.01). The therapeutic effect of EA was attenuated after inhibiting the release of the exosome.

**Conclusion:**

Our results indicated that EA improves intestinal motility in STC rats by transporting of exosomal miR-34c-5p targeting the SCF/c-Kit signaling pathway.

## 1. Introduction

Slow transit constipation (STC) is a dysfunctional intestinal disease with high morbidity worldwide [1]. The clinical manifestations of STC are the reduced frequency of defecation, hard feces, abdominal pain, and abdominal distension [2]. The pathogenesis of STC is decreased intestinal motility and colonic mucus secretion [3]. STC can be induced by an unhealthy diet, living habits, mental factors, and metabolic disorders [4]. Constipation recurs even after medication treatment. Recurrent STC damages patients' physical and psychological health and reduces their quality of life [5]. Although anticonstipation medications relieve constipation temporarily, long-term medication use may result in adverse effects, including diarrhea and abdominal distension [6]. In addition, many patients are not satisfied with the current medications [7]. Therefore, clinicians must find highly effective and affordable treatments with few adverse effects for managing constipation and explore the mechanisms of therapies for constipation.

The interstitial cells of Cajal (ICC) are a kind of specialized gut pacemaker cells found in the colon, bladder, and heart. ICC is closely related to gastrointestinal motility and can emit pacemaker potential [8]. Therefore, modulating ICC in the colon may be a potential therapy to promote intestinal motility when suffering constipation. Receptor tyrosine kinase (c-Kit) and stem cell factor (SCF) regulate the proliferation of ICC [9]. MicroRNA (miRNA) is single-stranded noncoding RNA with a length of 20–25 nt [10]. miRNA regulates genes by a specific sequence through combining with 3′-untranslated regions in a target messenger RNA (mRNA) [11]. miRNA plays an important role in diagnosing and treating gastrointestinal diseases [12]. An exosome is an extracellular vesicle with a diameter of 30–150 nm [13]. It has been reported that exosomal miR-34c-5p regulates ICC in STC model rats by targeting SCF. Besides, electroacupuncture (EA) can regulate exosomes, exosomal miRNAs, and exosomal circRNAs [14, 15].

Acupuncture is a traditional Chinese medicine (TCM) treatment with a long history of clinical use. EA treatment is a combination of acupuncture and electric stimulation. EA was proved to regulate intestinal motility with multiple targets [16]. Our previous experiments revealed that EA recovered the structure of ICC and regulated the expression and methylation of glial cell-derived neurotrophic factor (GDNF) in STC rats [17]. However, whether the mechanisms of intestinal motility recovery affected by EA are related to the mediation of exosomal miRNAs needs further exploration. Therefore, a rat model in which STC was induced with loperamide was used to investigate the mechanism by which EA promotes recovery through exosome-mediated miR-34c-5p.

## 2. Materials and Methods

### 2.1. Animals

Fifty SPF male Sprague-Dawley rats (220 ± 20 g) were purchased from Hunan SJA Laboratory Animal Co., Ltd. License number is SYXK2019-0004. All animal experiments were performed at the Animal Experimental Center of Hunan University of Chinese Medicine in a pathogen-free environment. All rats were fed with free water and food intake in a standardized environment (22 ± 2°C; 50 ± 10% relative humidity; 12 hours automatic light/dark cycle). The protocol of these experiments was approved by the Animal Ethics Committee of Hunan University of Chinese Medicine (approval number LL2021091502).

### 2.2. Animal Allocation and Induction of STC

Fifty rats were randomly divided into five groups (*n* = 10 each): a control group, STC, STC + EA, STC + MOS, and STC + EA + GW4869 groups.

Loperamide hydrochloride capsules were used to induce constipation, as previously described [18, 19]. These loperamide hydrochloride capsules were manufactured by Xian Janssen Pharmaceutical Ltd. and were purchased from the Pharmacy of the First Affiliated Hospital of Hunan University of Chinese Medicine, Changsha, China, with OTC No. H10910085. After one week of adaptive feeding, rats of STC, STC + EA, STC + EA + GW4869, and STC + MOS groups were subjected to loperamide hydrochloride (15 mg/kg/day, 10 mL/kg, b.w., in 0.9% sodium chloride) by intragastric administration once daily for seven days, with the dose was doubled on the first day. The control group was treated with 0.9% sodium chloride by gavage at 10 mL/kg per day for seven days.

### 2.3. Acupuncture and Drug Treatments

After the model was established, rats in the STC + EA and STC + EA + GW4869 groups received EA for 20 min once a day for 14 days. Rats were bound on a self-made fixator, and the abdomen and waist of rats were shaved to expose the skin's surface. The skin areas corresponding to acupoints were disinfected with 75% alcohol. Then, experimenters inserted acupuncture needles (made by Huatuo, Suzhou Medical Supplies Co., Ltd., Φ0.25 × 25 mm) into ST25 (Tianshu, situated in the abdomen, levelled with the navel, 5 mm next to the anterior midline) and BL25 (Dachangshu, located in the lumbar, under the fourth lumbar spinous process, next to 5 mm). After that, experimenters connected acupuncture needles to an SDZ-V electroacupuncture instrument using a dilatational wave with an electric frequency 2/15 Hz and an intensity of 0.5∼1 mA (an electric current intensity which led to a slight limb twitch was selected). An hour before EA treatment, rats in the EA + GW4869 group were intraperitoneally injected with exosome release inhibitor GW4869 (MedChemExpress, USA) at a 1 mg/kg dose every other day. GW4869 was dissolved in 10%DMSO. In addition, rats in the control and STC groups were immobilized on fixators in the same way for 20 minutes once a day for 14 days. Rats in the STC + MOS group were treated with mosapride by intragastric administration at a 2 mg/kg dose once a day for 14 days.

### 2.4. Evaluation of Excretion Behaviors

At the end of the induction period and on the 7^th^ and 14^th^ day after treatment, the fecal number and Bristol scores of all rats were observed. The rat metabolism cage was used to collect 24 hours of defecations, and the numbers of stool pellets were recorded. The Bristol stool scale score was used to assess stool characteristics [20].

### 2.5. Analysis of Intestinal Transit Rate

The intestinal transit rate was measured according to the method published in the literature [21]. The intestinal motility of rats was assessed through the intestinal propelling movement of black ink. After treatment, five rats in each group were administered 2 mL of black ink by intragastric administration. Thirty minutes later, rats were sacrificed, and the intestines were taken out. The tapeline measured the distance covered by the ink and the length from the pylorus to the ileocolic part. The formula for the intestinal transit rate was as follows: intestinal transit rate (%) = (distance covered by black ink ÷ the length from pylorus to ileocolic part) × 100%.

### 2.6. Hematoxylin and Eosin (H&E) Staining

Colonic tissues were fixed in a 4% paraformaldehyde and dehydrated with 100% alcohol and dimethylbenzene. Then, these were embedded in paraffin and sectioned into 5 *μ*m slices. Then, colonic sample slices were stained using hematoxylin and eosin (H&E) staining, following the standard method. Finally, we used light microscopy (Nikon Instruments Co., LTD, Japan) to observe the morphological characteristic of colon tissue slices.

### 2.7. Immunohistochemistry

Our study determined the expression of SCF and c-KIT protein by immunohistochemistry staining. Briefly, the colonic sample slices were prepared using the same way of H&E staining. Paraffin sections of colonic slices were dewaxed and antigens were repaired. The colonic slices were treated with 3% hydrogen peroxide and 5% normal goat serum. Primary antibody rabbit polyclonal anti-c-KIT antibody (diluted 1 : 200, Affinity Biosciences, USA) and rabbit polyclonal anti-SCF antibody (diluted 1 : 200, Bioss, China) were added separately into the colonic slices. After rinsing the slices, streptavidin was added. The slices were again rinsed, and DAB chromogenic solution was added. Finally, the slices were redyed with hematoxylin. After these were sealed, they were photographed using a microscope with 200 times the field of vision. The ratio of c-Kit-positive cells to total cells and the ratio of SCF-positive area to the total area were analyzed by using ImageJ software.

### 2.8. Exosome Isolation and miRNA Chip

The circulating exosomes were isolated from rat serum using ultracentrifugation (CP100MX, Hitachi, Japan). The rat blood samples were placed in a centrifugal tube and centrifuged at 2,000 × *g* at 4°C for 30 min. Then, the supernatant was transferred into a new centrifugal tube and centrifuged again at 10,000 × *g* for 45 min at 4°C. Next, the supernatant was filtered through a 0.45 *μ*m membrane, and the filtrate was centrifuged at 100,000 × *g* for 70 min at 4°C. Finally, the supernatant was removed and deposits were resuspended in cold PBS. The deposits were centrifuged at 100,000 × *g* for 70 min at 4°C. After the supernatant was removed, the pellet composed of exosomes was resuspended in 100 *μ*L of PBS.

### 2.9. Exosome Characterization

After the exosome samples were dripped onto a copper web, uranyl acetate was dripped onto the copper web for negative staining. The morphology of exosomes was observed using a transmission electron microscope (TEM).

The grain diameter and concentration of exosome were quantified using nanoparticle tracking analysis (NTA). The exosomes samples were diluted using a PBS buffer, using polystyrene particles to adjust the system and software.

### 2.10. RT-qPCR

RT-qPCR measured the expression of miR-34c-5p in colonic tissues and serum exosomal miR-34c-5p. Total RNA of colonic tissues was isolated by using TRIZOL reagent. The Exosome RNA Isolation Kit extracted the total RNA of exosomes, and miRNA was purified by the miRNeasy Mini kit from the total RNA of exosomes (217004, Qiagen, Dusseldorf, Germany). Reverse transcription of miRNA was carried out using First Strand cDNA Synthesis Kit (Yeasen; Shanghai, China). Then, PCR amplification reaction was conducted with the following cycle parameters: 95°C for 5min and 40 cycles at 95°C for 15 s and 60°C for the 60 s. The expression of miR-34c-5p was standardized to the rat U6 gene in tissue and miR-39 in serum. The relative expression was analyzed using 2^−ΔΔCt^. The following primer sequences used: miR-34c-5p (forward) AGGCAGTGTAGTTAGCTGATTG, (reverse) CAGTGCAGGGTCCGAGGTAT-3′; U6 (forward) CTCGCTTCGGCAGCACA, (reverse) AACGTTCACGAATTTGCGT; miR-39 (forward) AGCCCGTCACCTGGTGTAAATC, (reverse) GTCGTATCCAGTGCAGGGTCCGAGGTATTCGCACTGGATACGACCAAGCT.

### 2.11. Statistical Analysis

Data were presented as means and standard deviations. The SPSS 25.0 software (IBM Corporation, Armonk, USA) was used to analyze statistics. GraphPad Prism 9.2.0 (La Jolla, CA, USA) was used to create diagrams. One-way analysis of variance (ANOVA) with least-significant difference (LSD), post hoc test was used to examine statistical differences. The *P* value of <0.05 was deemed statistically significant.

## 3. Results

### 3.1. Regulation of EA on miR-34c-5p in Serum Exosomes and Colonic Tissues

**Figure 1:**
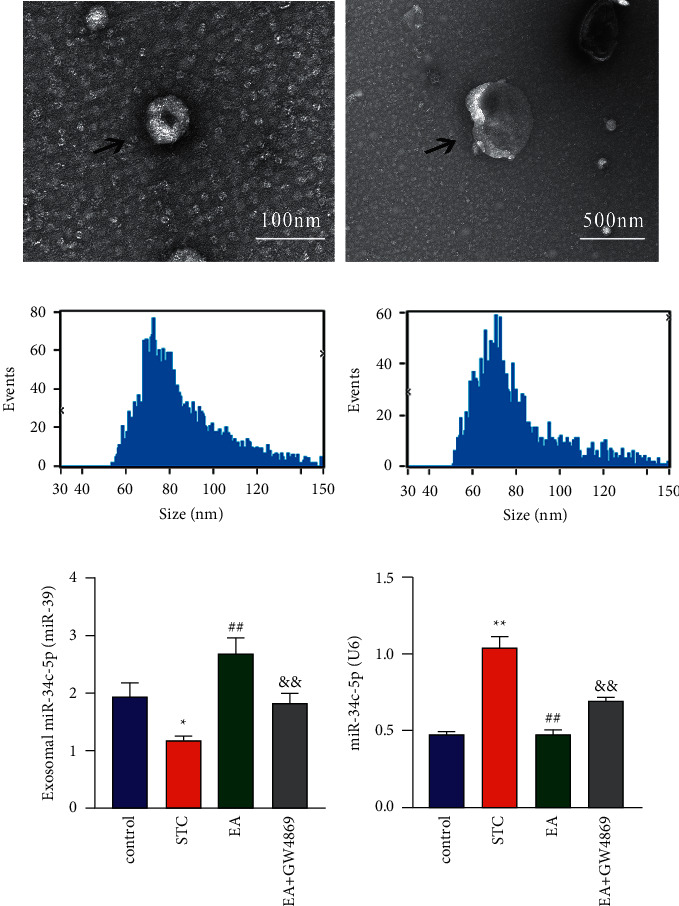
EA modulated the expression of serum exosomal miR-34c-5p and colonic miR-34c-5p. (a) Serum exosomes (arrows) were observed under a TEM, bar = 100 nm and 500 nm. (b) The particle size was measured using NTA. (c) RT-qPCR was used to measure the relative expression level of exosomal miR-34c-5p with miR-39 used as the internal reference. (d) RT-qPCR detected the relative expression of miR-34c-5p in colonic tissues; ^*∗*^*P* < 0.05 and ^*∗∗*^*P* < 0.01 compared with the control group; ^##^*P* < 0.01 compared with STC group; ^&&^*P* < 0.01 compared with STC + EA group.

To determine the probable correlation of miR-34c-5p in serum exosomes and colonic tissues after EA treatment, STC model rats were induced and exosomes were extracted from the rat serum. The ultracentrifugation method was applied to separate exosomes from the rat serum. TEM and NTA were conducted to confirm exosomes shape and particle size after isolation. TEM showed that the extract comprised cup-shaped vesicles with a monolayer membrane structure (Figure 1(a)). NTA demonstrated that most exosome particle sizes were 50–140 nm (Figure 1(b)). The results of TEM and NTA confirmed the characteristics of the exosome. GW4869 was used to detect if exosomal miR-34c-5p is related to the effect of EA. RT-qPCR was used to measure the expression of miR-34c-5p in serum exosomes and colonic tissues. The expression of exosomal miR-34c-5p was significantly downregulated in the STC group compared with the control group. At the same time, its upregulation was detected after EA treatment compared with the STC group, while it was decreased in the STC + EA + GW4869 group compared with the STC + EA group (Figure 1(c)). The expression of miR-34c-5p in colonic tissues was significantly upregulated in the STC group compared with the control group; it was downregulated after EA treatment compared with STC group, and it was increased in the STC + EA + GW4869 group compared with the STC + EA group (Figure 1(d)).

### 3.2. Exosomal miR-34c-5p Took Part in the EA Effect by Relieving Constipation and Improving Intestinal Motility

**Figure 2:**
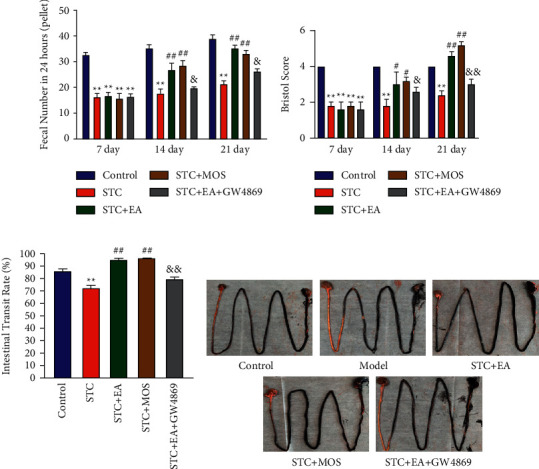
Exosomal miR-34c-5p took part in the EA effect by relieving constipation and improving intestinal motility. (a) Fecal number in 24 hours at 7, 14, and 21 days. (b) Bristol score at 7, 14, 21 days. (c) Intestinal transit rate, and (d) examples of the intestine and black ink trace for all groups. ^*∗∗*^*P* < 0.01 compared with the control group; ^#^*P* < 0.05, ^##^*P* < 0.01, compared with the STC group; ^&^*P* < 0.05, ^&&^*P* < 0.01 compared with the EA group.

The stool number, intestinal transit rate, and Bristol score were observed to measure the effectiveness of EA in promoting intestinal motility in STC model rats. After a week of modelling, the stool number and Bristol score were markedly lower in the loperamide-induced group than those in the control group, demonstrating that rats were successfully made to constipate by loperamide (Figures 2(a) and 2(b)). After one and two weeks of treatment, EA and mosapride significantly increased stool number and Bristol score as compared to those in the STC group (Figures 2(a) and 2(b)). The stool number and Bristol score were markedly lower in the STC+EA+GW4869 group than those in the STC+EA group (Figures 2(a) and 2(b)). However, there is no significant difference in the stool number and Bristol score between the STC + EA group and the STC + MOS group. Besides, there is no significant difference in the stool number and Bristol score between the STC group and the STC + EA + GW4869 group.

Compared with the control group, the intestinal transit rate was significantly lower in STC group than that in the control group (Figures 2(c)) and 2(d)). In contrast, the STC + EA group and STC + MOS group had a higher intestinal transit rate than that of the STC group (Figures 2(c)) and 2(d)). Besides, the intestinal transit rate was markedly lower in STC+EA+GW4869 group than that in the STC+EA group (Figures 2(c) and 2(d)). There is no significant difference in the intestinal transit rate between the STC + EA group and the STC + MOS group and between the STC group and the STC + EA + GW4869 group.

### 3.3. Exosomal miR-34c-5p Took Part in the EA Effect through Recovering the Colonic Histological Structure

In this study, H&E staining was used to explore the colonic mucosal histology. After two weeks of treatment, colonic tissues were histologically examined to detect whether EA could influence the histology of the colonic tissues.

**Figure 3:**
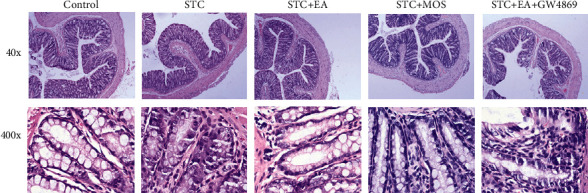
Exosomal miR-34c-5p took part in the EA effect through recovering the colonic histological structure. H&E-stained colonic tissues for all groups were observed at 100x and 400x.

In the STC group and the STC + EA + GW4869 group, the colonic mucosal surface of STC rats was uneven with hyperemia, and swelling and gland atrophy. Goblet cells were decreased. Inflammatory cells infiltrated the lamina propria. The muscle layer was thinner, and muscle cells showed vacuolar degeneration in the STC group (Figure 3). In STC + EA and STC + MOS groups, these histological changes were markedly ameliorated (Figure 3).

### 3.4. Exosomal miR-34c-5p Took Part in the EA Effect through the Proliferation of ICC and the Expression of the SCF

**Figure 4:**
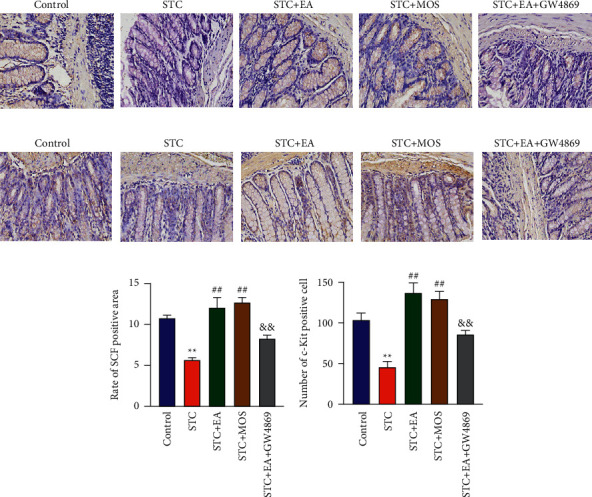
Exosomal miR-34c-5p took part in the EA effect through the proliferation of ICC and the expression of SCF. (a) Immunohistochemical staining of ICC (brown). The nuclei were visualized using hematoxylin (bluish-violet). Magnification: ×200. (b) Immunohistochemical staining of SCF (brown). The nuclei were visualized using hematoxylin (bluish-violet). Magnification: ×200. (c) The rate of the SCF-positive area. (d) The number of ICC in colonic tissues. ^*∗∗*^*P* < 0.01 compared with the control group; ^##^*P* < 0.01 compared with the STC group; ^&&^*P* < 0.01 compared with the EA group.

The expressions of SCF and c-Kit protein in colon tissues of STC rats in the STC group were significantly lower than those in the control group (Figures 4(c) and 4(d)). Compared with the STC group, STC rats in the STC + EA group and STC + MOS group had significantly higher SCF and c-Kit protein expressions in the colon (Figures 4(c) and 4(d)). Besides, cmpared with the STC+EA group, STC rats in the TC+EA+GW4869 group had markedly lower SCF and c-Kit protein expression in the colon (Figures 4(a)–4(d)).

## 4. Discussion

In our experiments, the stool number, intestinal transit rates, and Bristol scores were upregulated after STC model rats received EA and mosapride, demonstrating that EA is as effective as mosapride in improving intestinal motility and relieving constipation in this model. Based on the results of H&E staining, we confirmed that EA promoted the recovery of colonic tissue structures effectively. We believe that EA improved intestinal motility by activating the SCF/c-Kit signaling pathway based on immunohistochemical staining results. We determined whether EA influenced the transmission of exosomal miR-34c-5p and the release of exosomes. We found that serum exosomal miR-34c-5p expression was higher in the EA group than in the STC group. In comparison, the expression of miR-34c-5p in colonic tissues in the EA group was lower than that in the STC group. These results indicate that EA stimulates serum exosomal miR-34c-5p expression and inhibits colonic miR-34c-5p expression in STC model rats. We speculated that EA might inhibit the miR-34c-5p transport from serum to colon tissues.

Constipation is considered a primary gastrointestinal symptom worldwide. STC is a common subtype of chronic constipation, which features a lack of intestinal motility. Currently, laxatives are one of the most commonly prescribed medications for constipation [1]. However, laxatives may lead to side effects, including cramping, diarrhea, and electrolyte disorders [22]. Therefore, many patients seek complementary and alternative medicine to treat constipation [23].

Since the current intensity and frequency of EA can be uniform, we choose EA to treat STC model rats in these experiments. According to the TCM theory, back-shu and front-mu acupoints were two of the most classical acupoint selection methods. The back-shu acupoints “Dachangshu” (BL25) and front-mu acupoints “Tianshu” (ST25) were selected for this research. “Dachangshu” (BL25) is located at the bladder meridian of foot-taiyang, and EA stimulation can regulate gastrointestinal function [24]. “Tianshu” (ST25) is located in the stomach channel of foot-yangming, and it has many functions, such as treating gastrointestinal diseases and gynecopathy [25]. In addition, a previous clinical trial confirmed that EA at “Tianshu” (ST25) and “Dachangshu” (BL25) improves spontaneous bowel movements [26].

Some experiments have been conducted to determine how EA influences STC. Zhu et al. showed that EA influenced STC by upregulated tryptophan hydroxylase and 5-hydroxytryptamine in the colonic tissues [27]. Jin et al. found that EA improved STC by modulating the balance of the microbiota-gut-brain axis [28]. Liang et al. found that EA improves STC by regulating the enteric nervous system and ameliorating the enteric neuron function in the colon [25, 29]. Our experiments further revealed the mechanism of EA in treating STC. Loperamide is a widely used antidiarrhea drug in clinic, which inhibits the release of acetylcholine and prostaglandin and inhibits intestinal mucus secretion [30]. An STC rat model can be induced by intragastric administration of loperamide [31].

The density, structure, and distribution of ICC are related to the initiation and conduction of gastrointestinal slow waves, which govern the motility of the gastrointestinal tract [32]. The density of ICC was significantly decreased in colonic tissues in patients with STC [33]. SCF is a multifunctional cytokine that is widely distributed in bone marrow, kidney, ovary, and colon [34], and c-Kit is a specific marker of ICC. The SCF/c-Kit signaling pathway plays an important role in promoting the regular development of ICC through activating tyrosinase [24]. Yin et al. found naringenin-induced laxative effects by increasing the SCF/c-Kit signaling pathway in STC model rats [35]. Published studies indicated that the expression of SCF and c-Kit gene was decreased in STC model rats, and the effectiveness of treatment for constipation can be blocked by inhibiting SCF/c-Kit pathways [36]. To summarize, the SCF/c-Kit signaling pathway is a promising therapeutic target for STC treatment [37].

Exosomes are extracellular vesicles with a 30–120 nm diameter, secreted by various cells through exocytosis [38]. The contents of exosomes were transported to target cells and were ingested by target cells. Exosomes can modulate the activities of target cells through mediating cell-to-cell communication [39]. Recently, exosomal miRNA received increasing attention because it affects the development and progression of diseases [40]. Exosomal miRNA ?deleted value=“targeted-”? regulated cell apoptosis, autophagy, proliferation, and inflammation [41]. Since the function of exosomal miRNA, exosomal miRNA are highly fit candidates for use as diagnostic biomarkers, drug carriers, and genetic information carriers [42]. There is evidence that miRNAs take part in the pathogenesis of STC [43–45]. The results of co-expression network analysis and RNA-seq analysis showed that co-expressed miRNA networks with specific genes and pathways are related to STC [46, 47]. It has been reported that exosomal miR-34c-5p reduced ICC viability by targeting the SCF/c-Kit pathway in the colon [48] and can restrain vascular smooth muscle cell multiplication and neointimal proliferation [49]. As a widely distributed miRNA, miR-34 can mediate differentiation [50], cell apoptosis [51], multiplication [52], ageing [53], and modulate various signal pathways, including transforming growth factor-*β* (TGF-*β*) pathway [51] and Wnt pathway [54]. Thus, we speculated that exosomal miR-34c-5p played an important role in regulating STC. The concentrations of exosomes and the expression of exosomal biomarkers were changed after EA treatment, demonstrating that EA treatment could regulate exosomes [55]. These experiments emphasized the trafficking function of exosomal miR-34c-5p to probe the mechanism of EA.

GW4869 is a neutral sphingomyelinase inhibitor without side effects and intraperitoneal injection of GW4869 inhibits the release of circulating exosomes [56, 57]. Since the EA regulatory effect on exosomal miR-34c-5p, we found that the effectiveness of EA was impaired after the injection of GW4869. These results of experiments demonstrated that the block of the release of circulating exosomes weakened the effect of the EA. Hence, the delivery of exosomal miR-34c-5p from serum to colonic tissues may be a key mechanism of the EA effect.

Overall, we indicated that EA effectively improved constipation, promoted intestinal motility, relieved pathological lesions of colonic tissues, and motivated the SCF/c-Kit signaling pathway through the exosomal delivery of miR-34c-5p in the loperamide-induced STC model rats.

## 5. Conclusion

The exosomes of serum in STC rats were observed. EA improved constipation and led to the recovery of normal colonic histology STC rats. Furthermore, EA regulated miR-34c-5p in serum exosomes and colonic tissues; miR-34c-5p could target the SCF/c-Kit signaling pathway in colonic tissues. The effectiveness of EA was impaired by exosome inhibitor. Thus, EA could increase intestinal motility by regulating exosomal miR-34c-5p targeted the SCF/c-Kit signaling pathway.

## Data Availability

The datasets used and analyzed during the current study are available from the corresponding author on reasonable request.
